# Reversal of childhood idiopathic scoliosis in an adult, without surgery: a case report and literature review

**DOI:** 10.1186/1748-7161-4-27

**Published:** 2009-12-15

**Authors:** William J Brooks, Elizabeth A Krupinski, Martha C Hawes

**Affiliations:** 1 Restorative Care Foundation, Kansas City, MO 64152, USA; 2 Department of Radiology, University of Arizona, Tucson AZ 85724, USA; 3 Division of Plant Pathology and Microbiology, School of Plant Sciences, University of Arizona, Tucson AZ 85721, USA

## Abstract

**Background:**

Some patients with mild or moderate thoracic scoliosis (Cobb angle <50-60 degrees) suffer disproportionate impairment of pulmonary function associated with deformities in the sagittal plane and reduced flexibility of the spine and chest cage. Long-term improvement in the clinical signs and symptoms of childhood onset scoliosis in an adult, without surgical intervention, has not been documented previously.

**Case presentation:**

A diagnosis of thoracic scoliosis (Cobb angle 45 degrees) with pectus excavatum and thoracic hypokyphosis in a female patient (DOB 9/17/52) was made in June 1964. Immediate spinal fusion was strongly recommended, but the patient elected a daily home exercise program taught during a 6-week period of training by a physical therapist. This regime was carried out through 1992, with daily aerobic exercise added in 1974. The Cobb angle of the primary thoracic curvature remained unchanged. Ongoing clinical symptoms included dyspnea at rest and recurrent respiratory infections. A period of multimodal treatment with clinical monitoring and treatment by an osteopathic physician was initiated when the patient was 40 years old. This included deep tissue massage (1992-1996); outpatient psychological therapy (1992-1993); a daily home exercise program focused on mobilization of the chest wall (1992-2005); and manipulative medicine (1994-1995, 1999-2000). Progressive improvement in chest wall excursion, increased thoracic kyphosis, and resolution of long-standing respiratory symptoms occurred concomitant with a >10 degree decrease in Cobb angle magnitude of the primary thoracic curvature.

**Conclusion:**

This report documents improved chest wall function and resolution of respiratory symptoms in response to nonsurgical approaches in an adult female, diagnosed at age eleven years with idiopathic scoliosis.

## Background

Respiratory function within populations of patients with thoracic scoliosis, in general, is inversely correlated with curvature magnitude, with increasing impairment as Cobb angle increases [1-6]. However, substantial variation occurs among individual patients. At one extreme, cardiopulmonary disease secondary to idiopathic scoliosis (IS) has been reported to cause sudden death in young adults with 60- to 70-degree curves [7,8]. Other patients with similar curves may exhibit normal pulmonary function (PF) [5,9-12]. PF in patients with mild or moderate curves (<60 degrees) can range from normal to significantly impaired [5,11-22]. Thoracic kyphosis, normally ranging between 25-50 degrees, is reduced in some individuals ("hypo-kyphosis"), and this sagittal plane deformity together with reduced flexibility of the spine and thoracic cage may exacerbate pulmonary dysfunction [1,10,14,23-25]. Pectus excavatum, a depression of the sternum, is present in many scoliosis patients, and this condition by itself can contribute to PF deficiencies [26-28].

Even in patients without measurable impairment of VC in basal static conditions, reduced PF is often revealed during exercise tests [19,29-32]. A significant negative correlation has been shown between exercise capacity and Cobb angle from 10 degrees to 70 degrees in adolescent IS (AIS) patients [2]. Exercise capacity among a group of AIS patients with an average curve of 32.8 degrees and VC within normal limits was significantly reduced in comparison with a control group of subjects without spinal deformities [30]. The long-term impact of these impairments in scoliosis patients has not been examined, to date. However, in the general population, reduced VC and reduced exercise capacity are independent predictors of increased mortality [33-36]. Among 6213 men followed for 6.2 years, peak exercise capacity was a stronger predictor of early death than hypertension, smoking, and diabetes [36].

Impaired chest expansion resulting from chest wall deformity, rather than lack of physical activity or altered muscle tone, is thought to underlie reduced PF among patients with IS [1,37-42]. Evidence in support of this hypothesis includes the observation that PF defects characteristic of thoracic scoliosis can be induced in normal patients by immobilizing chest cage function with tape or corsets [43,44]. Conversely, PF improves in response to mobilization exercises designed to increase chest expansion [45]. Yet little information regarding long-term effects of impaired chest expansion in scoliosis patients is available. Among 195 AIS patients, evaluated at an average age of 42 years, chest expansion of <3.8 cm was found to be correlated with significantly reduced VC, recurrent respiratory infection, and shortness of breath [46]. Among a subset of this population of AIS patients evaluated after age >54 years (n = 57), 58% had chest expansion reduced to <2 cm, but PF was not measured [47]. However, in a separate study, among subjects in the general population (n = 48) evaluated at >54 years of age, only 15% had chest expansion reduced to <2 cm [48].

Patients with moderate to severe scoliosis diagnosed before puberty and present at skeletal maturity are at risk of curvature progression throughout life [46,49-52]. As a result of this increasing curvature, together with the normal effects of aging, women with scoliosis experience progressive loss in height beginning in early adulthood [53,54].

A previous study documented increased chest excursion and resolution of long-standing pulmonary symptoms in the subject of this case report [55]. The current report documents that, from ages 40-53 years, height increased by 2 cm, thoracic hypokyphosis improved, and the Cobb magnitude of the patient's longstanding thoracic curve was reduced. A preliminary report was presented at the May 2002 meeting of the International Research Society for Spinal Deformity in Athens, Greece [56].

## Case presentation

**Figure 1:**
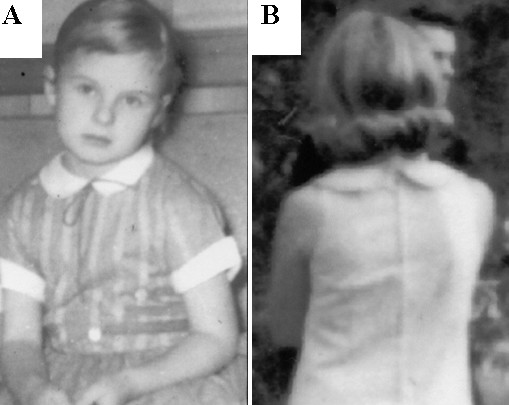
**Postural deformity as seen in casual photographs (A) age 6 years; and (B) age 16 years**.

The patient (MCH) is a female born 9/17/1952, the third of five children. Prenatal bleeding developed in the first trimester, and delivery (at full term) was breech and complicated by anoxia. Early development was normal, but a right inguinal herniorrhaphy was performed on 1/3/1959. By age 6.5 years an asymmetric posture [57] was apparent in casual photographs (Figure 1A). After puberty a marked torso deformity was evident (Figure 1B). From childhood through 1992 (age 40 years), symptoms included dyspnea at rest, increasing in severity and associated with nausea upon mild exertion or at high altitude. Recurrent respiratory infections with fever and deep chest cough (3-5 episodes per year, each lasting 3-10 weeks) also were present. The patient lived in a rural area, never suffered from asthma, never smoked, and avoided proximity to smokers due to sensitivity to secondhand smoke. No seasonal allergies were present during childhood and early adulthood. No pulmonary function tests were prescribed by treating physicians during this period. During childhood daily activities included participation in marching band, swimming, horseback riding and other farm chores but recurrent vertigo precluded gymnastics and other formal sports.

At age 11.7 years (pre-menarche), the patient was seen by her pediatrician in response to obvious torso deformity identified by an older sibling while horseback riding. There was no family history of scoliosis or other spinal deformities. Referral was made to an orthopedic surgeon (J. Stiles, Owensboro Daviess County Hospital, Owensboro KY, USA). The diagnosis was IS with a right thoracic Cobb angle of 45 degrees. A compensatory lumbar curve, thoracic hypo-kyphosis, and pectus excavatum were also present. Immediate spinal fusion surgery was strongly recommended but declined. Exercises taught during a 6-week period of outpatient training by a hospital resident physical therapist were prescribed as an alternative to surgery (Table 1). This program was essentially as outlined in Ponseti and Friedman [58] who described a regime of conservative treatment used in the long-term 'natural history' [46,47] study of 444 patients carried out at the University of Iowa (Iowa City, IA, United States). The authors stated that "*a great number of patients in this series received conservative treatment consisting of exercises designed to increase muscle strength and to correct postural imbalance. Tightness of the abdominal muscles at the side of the overhang was corrected by passive and active stretching. The patients were taught to shift their thorax into proper alignment with the pelvis*" ([58], p. 381).

**Table 1:** Outline of exercises and treatments, 1964-2005

Year	Exercises and Treatments
1964-1974:	Prescribed calisthenics, stretching, walking (≥ 30 minutes daily)

1974-1991:	Calisthenics, stretching, plus aerobics (biking, jogging) (60 min daily)

1991:	Deep tissue massage therapy (monthly 60-minute sessions, June-December)

1992-2001:	Daily home mobilization exercises (no strengthening or aerobic exercise)
	Outpatient psychological therapy (1992, 1993)
	Deep tissue massage therapy (fourteen 60-minute sessions)
	CMM (four sessions in 1994-1995; seven sessions in 1999-2000)

2001-2005:	Daily mobilization, strengthening, and aerobic exercise (40-50 min daily)
	Incentive spirometry (5 reps daily)

These exercises, in the current case, included sit-ups, side-to-side toe-touches, push-ups, 'bicycle' leg lifts, and straight leg raises (15-20 reps daily), plus side-bending and stretching in combination. Four recommended exercises were discontinued: (1) Hanging by the arms from a bar triggered immediate pain in the right inguinal region (where scar tissue from herniorrhaphy was present) and lower back, with loss of sensation in the right foot; (2) a posture exercise in which the back was flattened repeatedly against a wall triggered neck pain; (3) assisted neck stretches, which required the patient to bend her head to the side facilitated by a partner (parent), also were discontinued due to increased pain; (4) back bends from a prone position were discontinued when the supervising physical therapist noticed exacerbation of the torso asymmetry within 2-3 weeks of daily replication. The other exercises were included in a 30-40 minute regime carried out daily from 1964-1974. In 1974, in response to increased dyspnea while living at high altitude, a 30-40 min aerobic exercise routine was added to the daily routine and maintained through 1992. Depending on circumstances, this included a daily 10-mile round-trip hike to work, a 16-mile round trip bicycle ride to work, or a 2-mile jog at a local track. Yoga headstands and kicks also were added. Dyspnea persisted as a symptom despite these activities.

Deep tissue whole body massage was carried out monthly from June-December 1991 by a licensed massage therapist (C. Kotch, Tucson AZ, USA) in response to neck pain; fourteen additional sessions were carried out from December 1992-February 1996. The primary focus of treatment was mobilization of the right psoas, adhesions surrounding right inguinal scar tissue, chest wall rigidity and limited cervical spine range of motion. In February 1992 outpatient individual psychological therapy was initiated in response to psychological decompensation concurrent with intense multi-regional pain [55]. Daily strengthening and aerobic exercises were discontinued at this time and replaced with torso, abdominal and chest wall mobilization and respiratory exercises including 'loud vocalization' [59]. In April 1992 a noticeable improvement in torso appearance developed suddenly, manifested by spontaneous decrease in forward rotation of the right shoulder (Figure 1). This occurred in correlation with increased ability to turn the head to the side, decreased discomfort and a sensation of improved breathing.

In February 1993 the patient (MCH) initiated a collaboration with the first author (WJB, Faculty of Medicine, University of Arizona), for a study designed to evaluate and document any changes in signs and symptoms (e.g. respiratory function, chest wall morphology and excursion, Cobb angle). In addition, intervention in the form of comprehensive manipulative medicine (CMM) was carried out four times during 1994-1995 and on seven occasions during 1999-2000 [55,60]. This approach to the use of manipulative techniques significantly differs diagnostically from methods that employ spinal mis-alignment, postural imbalance, asymmetrical active range of motion, and/or asymmetrical passive range of motion as sufficient markers of musculoskeletal dysfunction [e.g. [61]]. CMM refers to the use of a broad spectrum of (in this case, direct action) manipulative medicine techniques employed for the purpose of restoring optimal available motion to the entire musculoskeletal system, including the cranium, as described [55,60]. A daily 40- to 60-min home mobilization exercise program (1993-2005) included sustained pressure applied to painful muscle spasms, isometric chest stretches (palms pressed together while expanding the rib cage), and manual traction using a home gym [56]. Incentive spirometry was added in 2001. Aerobic and strengthening exercise (sit-ups, abdominal crunches, bicycle, hiking, jogging, side kicks, weight lifting, etc) was re-incorporated into the daily regime in 2001. Body weight was stable at 62 ± 3 kg.

### Measurement of Chest Expansion, Torso Deformity, and Cobb Angle

The chest excursion and hemi-thorax size measurements were performed with a cloth tape measure referencing specific bony landmarks [48,55,62]. Hemi-thorax size is the circumferential distance around the hemi-thorax at maximum exhalation and maximum inhalation, and is expressed as mean and standard error of 30 replicates taken over a 2-day period. Measurement of height was carried out in the morning. Rib prominence measurements, given as mean and standard error of at least 10 replicates over a 3-day period, were based on the Adams forward bending test using a Bunnell scoliometer [63]. Intra-thoracic diameter was measured from lateral films, using a ruler placed between the anterior body of the T-7 vertebra and the posterior face of the sternum. Vertebral rotation was estimated according to the method of Nash and Moe [64].

To our knowledge, variability in Cobb angle measurement in aging adults has not been evaluated systematically. However, substantial inter-rater and interdisciplinary variability in Cobb angle measurement has been documented, especially in secondary curves and in cases where different end-points are selected for curvature classification [65-70]. Therefore Cobb measurement was done by two methods, a double blind study by radiologists at the treating institution (Method 1), and nonblinded readings by two scoliosis specialists at independent institutions (Method 2). All standing anterior-posterior (AP) radiographs and sagittal radiographs were made at the same facility by the same technician (W. Quirk, Senior Radiology Technician, University Medical Center, Tucson AZ), and were taken at maximum inhalation with the same instructions to the patient: "Stand up straight, take a deep breath, and hold." To provide blinded Cobb angle readings, as described by Goldberg et al. [66], the second author removed from the films all identifying information and any markings from previous measurements. An identifying number then was assigned arbitrarily to each set of radiographs taken at four-year intervals. Three readers, none of whom had seen the films previously or were known by the patient or the first author, were given the same set of films in random order. All three readers are board-certified by the American Board of Radiology, http://theabr.org/ and employed in The University of Arizona College of Medicine, Department of Radiology, Tucson AZ. Each recorded three readings for each film. Repeated measures analysis of variance was used to evaluate statistical significance of mean difference.

## Results

### Increase in height

The patient's height increased by 2 cm between 1990 (65.70 inches; 167.64 cm) and 2005 (66.5 inches; 169.67 cm).

### Improved pulmonary symptoms

Vital capacity in 1996, 2001, and 2005, respectively, was 1.6 liters (71% predicted), 3.97 liters (115% predicted), and 3.99. Relief from respiratory symptoms including dyspnea and recurrent respiratory infection, as reported previously [55], was maintained.

### Improvement in torso symmetry

**Figure 2:**
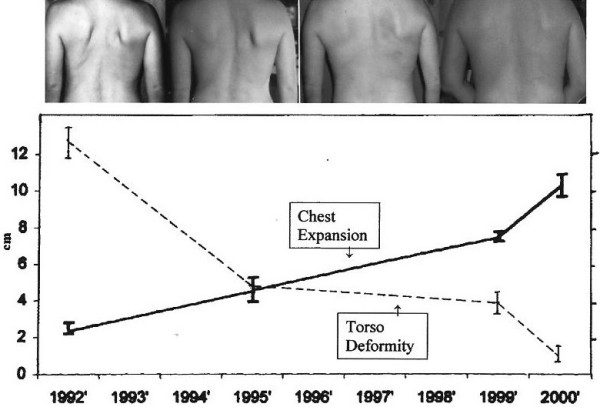
**Improved chest wall morphology and function**. Top: Clinical aspect of the deformity documented with photography. Photos (left to right) were taken in 1991, 1995, 1999, and 2002, respectively. Bottom: Inverse relationship between increased chest expansion (solid line) and reduced torso deformity (broken line). 'Chest expansion' (cm) was measured directly based on the difference in total chest circumference at minimum and maximum inhalation [48,62]; 'torso deformity' values (cm) reflect the difference between right and left hemi-thorax, at maximum inhalation. Each value represents mean and standard deviation from at least 30 measurements taken over a 48-h period, measured at intervals between 1991 (patient age: 39 years) through 2005 (patient age: 53 years, 2.1 years post-menopause).

In 1992, there was a 12 ± 2 cm difference between the left and right hemi-thorax at maximum inhalation, and a 10 ± 1 cm difference at maximum exhalation; by 2005, these differences were reduced to 2 ± 2 cm and 1 ± 1 cm, respectively (Figure 2). The rib prominence [61] was reduced from 18 ± 3 to 11 ± 2 degrees.

### Increase in sagittal plane Cobb magnitude

**Figure 3:**
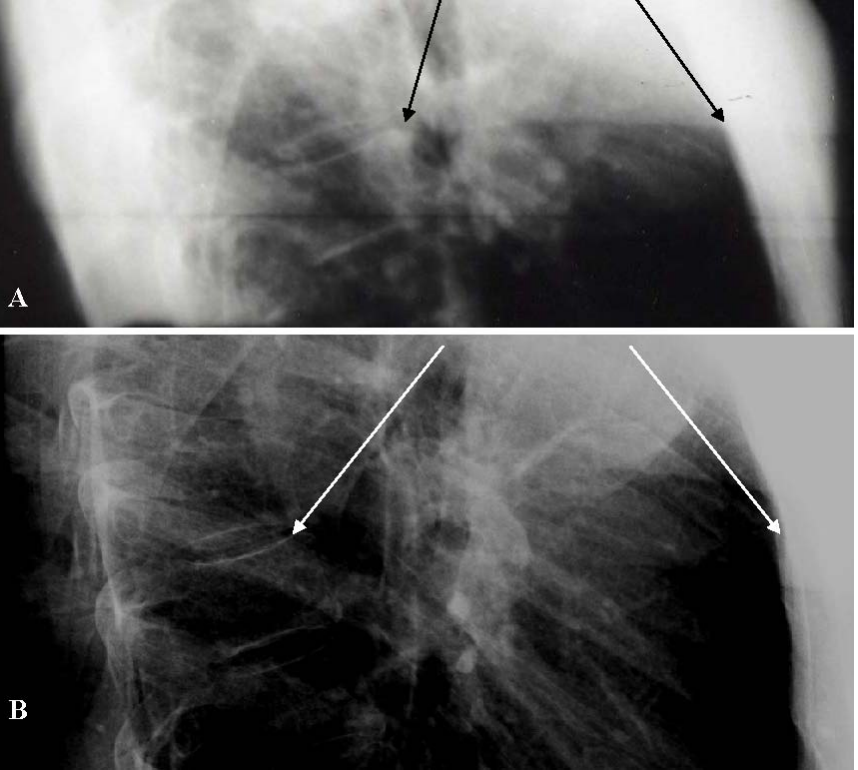
**Increased sagittal plane curvature**. Lateral radiographs of the thoracic spine, were taken in (A) 2001; and (B) 2005. Arrows indicate the anterior edge of T7 (left) and a bony landmark on the posterior edge of the sternum (right). Radiographs were taken by the same technician, with the patient in a standing position, at maximum inhalation, using the same machine (University Medical Center, Tucson AZ).

The Cobb angle of the sagittal thoracic curvature increased from 17 ± 2 degrees in 2001 (Figure 3A) to 33 ± 3 degrees in 2005 (Figure 3B). During the same period, the AP intra-thoracic diameter during maximum inhalation, as measured directly on a sagittal radiograph, increased from 8.0 cm (Figure 3A, white arrows) to 10.5 cm (Figure 3B, black arrows).

### Decrease in coronal plane Cobb magnitude

**Figure 4:**
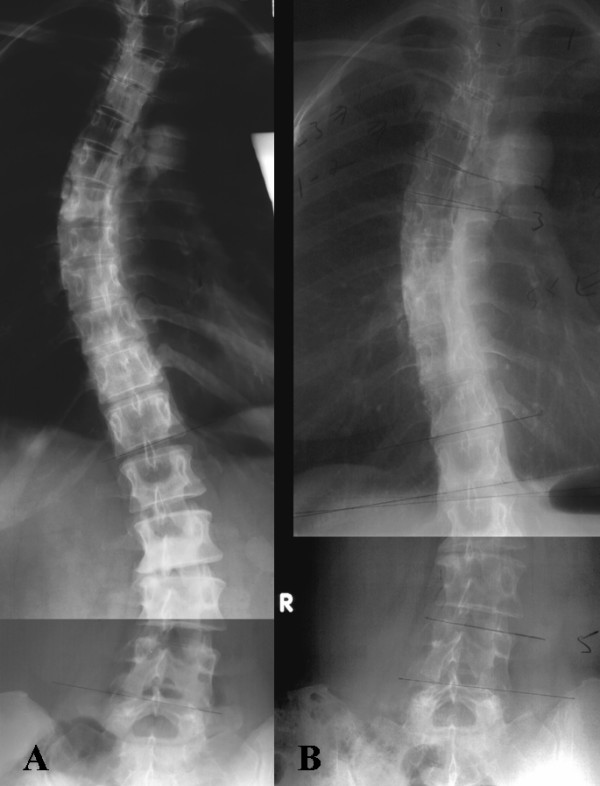
**AP standing radiographs in (A) 1990 and (B) 2005**.

From 1990 (Figure 4A) through 2005 (Figure 4B) the magnitude of Cobb angle for the primary thoracic curve declined by >10 degrees (Table 2). Grade I-II rotation of each apical vertebra was present [64].

**Table 2:** Cobb magnitude measurements, 1990-2005^1^

Vertebrae	1990	1994	1998	2001	2005
***Method #1***					

***T4-T12***	***47 *± *2***	***38 *± *2***	***34 *± *1***	***28 *± *2***	***24 *± *2***

L1-L4	26 ± 1	19 ± 2	17 ± 1	13 ± 1	10 ± 2

***Method #2*^2^**					

**T4-T11**	**45**	**39**	**35**	**34**	

**T4-T10**					**33**

T12-L4	30	24	22	22	

T11-L3^3^					23

***Method #2*^4^**					

**T4-T11**	**45**	**38**			**34**

**T5-T11**			**36**		

T12-L4	27				22

^1^The values for the primary thoracic curvature are provided in bold-faced type. Method #1: values for Cobb angle represent means and standard deviation from triplicate readings by each of three readers independent of this study, according to protocols defined by Goldberg et al. [66]. Cobb magnitude mean values for the primary and secondary curvatures, in 1990, were statistically distinct from the mean values in 2005 (p < 0.001). Method #2: Two readers from independent institutions carried out non-blinded readings from film copies provided by the authors.

^2^Method #2, measurements by Reader #1.

^3^L2-L3 intervertebral space narrowing and degeneration with spur formation. The character of the primary thoracic curve from 1990-2005 was judged to be altered, from eight included vertebrae to seven (T4 - T11 to T4-T10). The altered endpoints are provided.

^4^Method #2, measurements by Reader #2. The character of the primary thoracic curve from 1990-2005 was judged to be altered, from eight included vertebrae to seven (T4-T11 to T5-T11). The altered endpoints are provided.

## Discussion

The negative impact of thoracic spinal deformity on respiratory function was recognized and described in ancient times by Hippocrates [1,59]. Current studies among children and adults with thoracic scoliosis whose Cobb angle ranged from 30-80 degrees have revealed measurable pulmonary deficits manifested as impaired vital capacity, reduced exercise capacity, cardiac hypertrophy and symptoms including dyspnea and recurrent respiratory infections [1-25,29-42,46]. Yet in recent years some spine surgeons have argued that, with the exception of cases diagnosed before the age of five years and with a Cobb angle of >100 degrees, IS patients suffer from '*no functional limitations*' and that, moreover, the improvement of *'appearance and deformity with all its social and psychological deprivation is the only indication for treatment' *[71]. The stated goal of such treatment is '*to restore acceptability and help mitigate future social and psychological disadvantage*' [72], not to restore or maintain health and function. Even routine PF testing for scoliosis patients prior to surgery is described as *'controversial*' [73]. As long ago as 1964, however, Mankin and coauthors [21] noted, *"Most physicians think that IS is a 'cosmetic' problem and that its major effect is to deform an individual with no harm to the internal organs. It is apparent from this study that appreciable pulmonary deficits do occur but are easily detected by simple spirometry." *Chest cage rigidity, which may contribute to PF deficits in scoliosis patients, increases with age, could in part account for the observation that respiratory failure can occur in adult IS patients even when there is no progression of the curvature [74,75]. The possibility that undiagnosed PF deficits contribute to psychological distress found to be prevalent among juvenile, adolescent, and adult IS patients, remains unexplored [76-80].

In patients who undergo spinal fusion surgery, VC does not improve in correlation with improved Cobb angle [1,9-12], and among surgery followup studies published since 1941 the impact of spinal fusion on chest excursion has never been included as an outcome measure [81]. By contrast, significantly improved VC and increased chest expansion occurred in several hundred scoliosis patients within four weeks in response to an inpatient physiotherapeutic approach [45,82]. This approach now has been adapted successfully for outpatient treatment strategies [83,84]. Recent clinical studies reveal success in reducing pain and improving Cobb angle with exercise, manipulation, and other nonsurgical approaches in patients, including adults [85-90]. Rapid improvement of pectus excavatum severity in an adult, in correlation with individualized physical therapy, also has been documented [91]. The results from these studies, together with the current case report, indicate that despite popular beliefs [71] a reduction in clinical signs and symptoms of scoliosis that impair quality of life and function can be achieved in response to specific physical interventions. Investigations of non operative methods in the treatment of adult scoliosis are warranted [92-98]. Based on the known biological and biomechanical processes associated with progression of scoliosis, application of such methods can be predicted to be most effective in halting or reversing lateral and torsional deformity of the spine when employed early in the disease process [99,100]. School screening programs have been found to be an effective method to detect and diagnose scoliosis in its earlier stages, and can play a key role in such research [101].

## Conclusions

Stable improvement in chest wall symmetry and resolution of long-standing respiratory symptoms in an adult with pectus excavatum, thoracic scoliosis and hypokyphosis, has not previously been reported. Improvement in magnitude of Cobb angle occurred in parallel with the observed changes in function.

## Competing interests

The authors declare that they have no competing interests.

## Authors' contributions

MCH, EAK, and WJB each participated in the design and writing of this manuscript. EAK recruited the team of experts for Cobb angle readings of serial followup radiographs at the treating institution, supervised the blinded measurement process, and carried out statistical analysis of the data. MCH (the patient in this case report) carried out the literature search. All authors read and approved the final manuscript.

## Consent

Informed consent was obtained from the patient for publication of this case report and accompanying images. A copy of the written consent is available for review by the Editor-in-Chief of this journal.
